# Sensor-Based Assessment of Upper-Limb Motor Control in Children and Adults Using Two-Dimensional Circular Target Tracking

**DOI:** 10.3390/s26175344

**Published:** 2026-08-24

**Authors:** Yohan Song, Jihun Kim, Jongho Lee, Jaehyo Kim

**Affiliations:** 1 Department of Human Ecology & Technology, Handong Global University, 558, Handong-ro, Heunghae-eup, Buk-gu, Pohang-si 37554, Gyeongsangbuk-do, Republic of Korea; 2 Glocal University 30 Project Group, Handong Global University, 558, Handong-ro, Heunghae-eup, Buk-gu, Pohang-si 37554, Gyeongsangbuk-do, Republic of Korea; 3 Department of Electrical Engineering, Faculty of Engineering, Fukuoka Institute of Technology (FIT), 3-30-1 Wajiro-higashi, Higashi-ku, Fukuoka 811-0295, Japan; 4 Department of Mechanical & Control System Engineering, Handong Global University, 558, Handong-ro, Heunghae-eup, Buk-gu, Pohang-si 37554, Gyeongsangbuk-do, Republic of Korea

**Keywords:** sensor-based human motion analysis, upper limb assessment, visuomotor tracking, feedback control, feedforward control, predictive tracking, motor development, human–computer interaction

## Abstract

Portable sensor-based assessment of human motion is important for monitoring motor development and informing the design of rehabilitation applications. This study investigated upper limb motor characteristics during two-dimensional circular target tracking across different age groups and speeds. Fifty-one participants were divided into three groups: lower-elementary children, upper-elementary children, and adults. Using a tablet- and stylus-based input device suitable for human–computer interaction systems, participants performed the tracking task at three speeds. Each trial included relatively feedback-dominant target-visible segments and relatively feedforward-dominant temporarily target-invisible segments. The latter imposed greater demands on predictive tracking because current target-related visual information was unavailable. A participant-level Tracer Error Ratio was calculated as the target-invisible positional error divided by the target-visible positional error. The two child groups showed similar ratios across all speeds, whereas adults showed lower ratios than either child group. As tracking speed increased, the ratio decreased in all groups, with the adult mean falling below 1 only in the high-speed condition. These findings indicate an adult-child difference in predictive tracking during temporary target disappearance but do not directly identify a specific cerebellar mechanism. This portable tablet-stylus approach may be useful for research on age group differences in upper limb target tracking performance.

## 1. Introduction

Human motion analysis has become an important research field for the objective assessment of motor behavior in healthcare, rehabilitation, sports science, ergonomics, and human–computer interaction. Recent advances in sensing technologies have enabled quantitative movement assessment outside specialized motion analysis laboratories. Data-driven computational approaches combined with sensing technologies have also expanded quantitative biomechanical assessment under various movement conditions [1]. In particular, portable and low-cost sensing platforms can provide accessible tools for evaluating motor development and motor impairment in school, clinical, rehabilitation, and home-based environments [2]. For upper limb assessment, coordinate-based tracking tasks are useful because they allow movement accuracy, timing, and control characteristics to be quantified from two-dimensional trajectory data.

Age-related differences in motor control are closely related to brain development, neuromotor maturation, sensory processing, muscle coordination, and physical growth. Understanding these differences is important for characterizing children’s motor development and for developing assessment methods that may later be extended to older adults or patients with neurological and developmental disorders. Therefore, quantitative sensor-based assessment may provide practical data for healthcare, longitudinal monitoring, and objective evaluation of motor function.

Human motor control is generally understood through feedback and feedforward control models of the brain [3,4]. A schematic representation of the feedback and feedforward motor-control framework, including the inverse model and the integration of feedback and feedforward motor commands, is provided in Figure S1. Feedback control uses sensory information to correct deviations between intended and actual movement, whereas feedforward control relies on predictive motor commands generated from internal models. Previous studies of reaching movements have shown that feedforward commands predict time-dependent trajectories, while feedback components correct movement errors based on sensory information regarding the current system state [5]. Other studies have demonstrated that feedforward commands and feedback responses can share internal models of arm dynamics, indicating that both processes are adjusted when the physical characteristics of the arm change [6]. In school-aged children, the balance between feedback and feedforward control changes during development as stored motor data, sensory correction, and predictive control mature [7,8].

Although reaching movements provide important information about motor control mechanisms, tracking movements are particularly useful for analyzing repetitive movement because movement error can be continuously observed while participants follow a moving target. Target tracking tasks that include both visible and invisible target segments provide a framework for comparing feedback-dominant and feedforward-dominant control. Temporarily invisible target paradigms have been used to investigate predictive representations of target motion, including developmental changes in predictive tracking during childhood [9]. When the target is visible, participants can use visual feedback to correct position errors. When the target becomes invisible, they must rely more strongly on feedforward prediction and the internal representation of the target trajectory.

The internal model of human motor control refers to neural mechanisms in the central nervous system that simulate the behavior of the motor system and the external environment. Internal models predict the outcomes of motor commands and help determine the commands required for specific tasks [10]. They include forward models, which predict future states based on the current state and motor commands, and inverse dynamics models, which calculate the motor commands needed to achieve desired state changes [11]. Visual stimulus response processing is not instantaneous; simple visual reaction times of approximately 300 ms have been reported in basic stimulus–response tasks [12]. Predictive internal model processing may therefore support tracking when current target-related visual information is delayed or temporarily unavailable [13]. These concepts help frame the interpretation of tracking during temporary target disappearance.

Target tracking movements are associated with the formation and modification of motor models in the cerebellum. Previous studies have shown that predictive models are formed through feedback and feedforward processes and that errors in these models are corrected during repeated movements [14,15]. Other studies have also examined responsiveness and error correction using ocular and manual target tracking tasks, visible–invisible paradigms, hand tracking tasks, and wrist tracking movements [8,9,16,17,18]. The cerebellum plays a key role in correcting errors while controlling speed and position [19,20], and feedback error learning theory proposes that feedback error signals contribute to continuous modification of cerebellar internal models during motor learning [21,22]. Within this framework, visual information about the target and the resulting tracking errors provide feedback signals for updating the internal model. As the internal model is refined through repeated tracking, it can generate predictive motor commands. Thus, if visual information about the target is not available, tracking relies more strongly on feedforward commands generated from the internal model. On this basis, tracking with and without available visual target information can be interpreted as feedback-dominant and feedforward-dominant, respectively. This process is fundamental to motor development from childhood to adulthood.

Previous circular tracking studies have compared feedback and feedforward control performance at varying target speeds and have quantified motor control characteristics using radius error, angular error, and angular velocity error [18]. Related studies have also analyzed children’s motor development using two-dimensional plane errors and investigated differences in motor performance between children and adults [23,24]. However, relatively few studies have directly used a portable sensor-based task to compare feedback- and feedforward-dominant upper limb tracking performance across age groups and multiple target speeds. A simple digital metric that summarizes the relationship between visible and invisible segment tracking errors may help characterize age-related differences in internal motor model use during upper limb control.

From a sensor engineering perspective, tablet- and stylus-based systems offer a practical platform for upper limb motion assessment. Such systems can record two-dimensional fingertip or stylus tip trajectories with minimal preparation, do not require external markers or camera calibration, and can be deployed easily in schools, clinics, rehabilitation facilities, and home-based environments. Compared with laboratory-based motion-capture systems, tablet–stylus platforms provide a simpler and more accessible method for repeated measurement of upper limb tracking performance. These characteristics are compatible with sensor-based human motion analysis and are relevant to developmental assessment, rehabilitation monitoring, adaptive human–computer interaction, and future human–robot interaction applications.

In the present study, we analyzed age-related upper limb motor control using the Tracer Error Ratio derived from circular target-tracking movements in a two-dimensional plane. The task involved multi-joint upper limb hand movements performed with a stylus-based input device on a tablet PC. It included visible target segments, which primarily reflected feedback-dominant control, and invisible target segments, which required greater reliance on feedforward prediction and internal representation of the target trajectory. Two-dimensional position errors were calculated from the recorded target and stylus coordinates. The Tracer Error Ratio was defined as the ratio of the mean positional error during invisible target segments to that during visible target segments and was used as a sensor-derived digital metric to compare feedback-dominant and feedforward-dominant tracking performance across age groups and target speeds. We hypothesized that this metric would reveal developmental differences in upper limb visuomotor control and demonstrate the potential of a portable tablet–stylus sensing platform for human motion analysis and future adaptive interaction systems.

## 2. Materials and Methods

### 2.1. Apparatus and Environment

**Figure 1:**
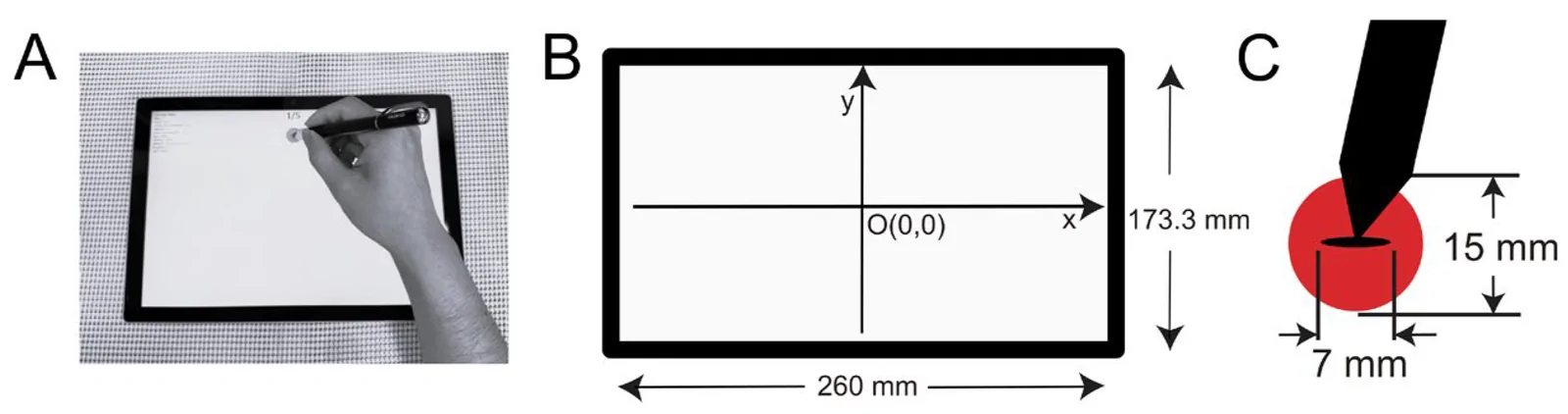
(**A**) Tablet PC screen, (**B**) display dimensions, and (**C**) dimensions of the target marker and stylus tip.

This study used a motor ability evaluation system based on a personal tablet computer (PC). The system recorded the target and stylus coordinates in real time during the task for subsequent evaluation of tracking performance. The device used in this study is shown in Figure 1.

The measured width of the stylus tip was 7 mm, and the diameter of the red target marker was set to 15 mm. A Microsoft Surface Pro 7 (QWF-0006; Microsoft Corporation, Redmond, WA, USA) with a 12.3 inch display, a 3:2 aspect ratio, and a resolution of 2736 × 1824 pixels was used. Based on the specified diagonal display size and aspect ratio, the display dimensions were calculated to be approximately 260.0 mm in width and 173.3 mm in height. The circular target trajectory had a radius of 62.5 mm, corresponding to a diameter of 125 mm.

### 2.2. Participants

This study included 17 younger elementary school children (5 males, 12 females, mean age 9.12 ± 0.90 years), 17 older elementary school children (5 males, 12 females, mean age 11.59 ± 0.69 years), and 17 adults (10 males, 7 females, mean age 22.65 ± 3.05 years). All the participants provided written consent before participating in the experiment, and written consent was obtained from the guardians of the children participating. Table 1 summarizes participant demographics. Adult hand dominance was assessed using the Edinburgh Handedness Inventory, while a modified version of the same inventory, adapted for children, was used to assess hand dominance in the child participants. The experimental protocol was approved by the Institutional Review Board (IRB) of Handong Global University (2023-HGUA021) and conducted in accordance with the Declaration of Helsinki.

### 2.3. Task Description

**Figure 2:**
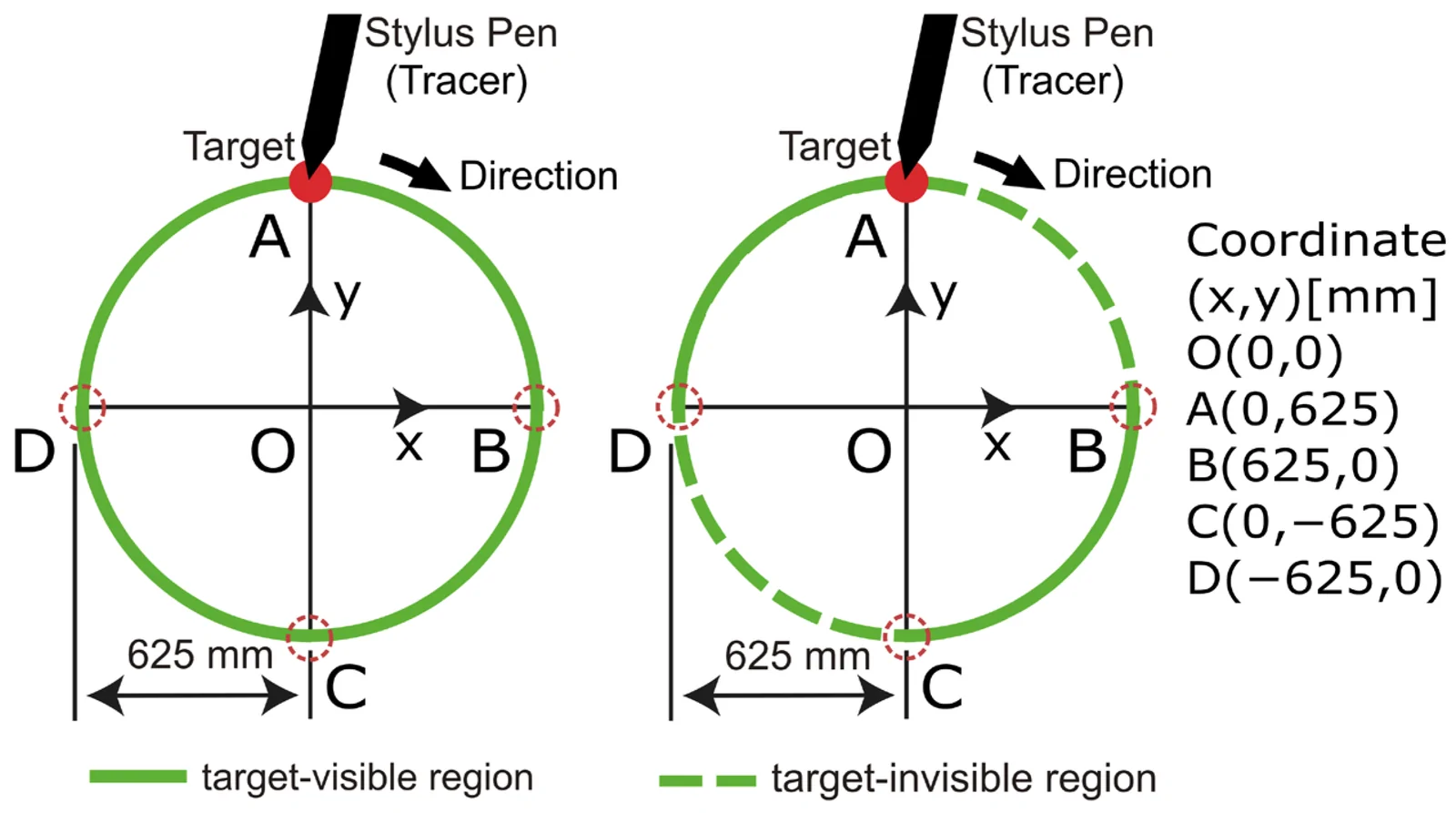
Schematic of the circular target tracking task for right-hand tracking. The (**left**) shows the target-visible cycle, and the (**right**) shows the cycle containing target-invisible segments. For left-hand tracking, the movement direction and target-invisible regions were mirror-reversed.

Participants performed target tracking exercises using a tablet PC while holding the prepared stylus in front of a desk. A portable tablet PC was placed on the desk with the display facing upward, showing a red dot as the target. The target’s trajectory was transparent and invisible. Participants placed the stylus on the red target dot (positioned at the top-center of the screen with a trajectory radius of 12.5 cm). Following a standby cue, participants moved the stylus along a circular path, following the target. Right-handed tracking proceeded clockwise, while left-handed tracking proceeded counterclockwise. The opposite rotation directions were selected to provide a mirror-reversed task geometry across the two hands, consistent with the symmetrical mirror mode directional arrangement described in bimanual circle drawing research [25]. All participants performed five repetitions at each of three speeds (slow, medium, and fast), progressing from slow to fast. The speed for slow tracking motion was 0.125 Hz (tangential speed: 0.049 m/s), for medium tracking motion was 0.25 Hz (tangential speed: 0.098 m/s), and for fast tracking motion was 0.5 Hz (tangential speed: 0.196 m/s). Each trial consisted of four cycles of the circular target-tracking motion, with two training cycles (Figure 2, left) and two test cycles, in which the target disappeared and reappeared (Figure 2, right). The target remained continuously visible during the first two cycles, and these cycles were analyzed as the target-visible condition. After completing the exercises with one hand, participants performed the same exercises with the other hand, reversing the rotation direction. The order of hand testing was randomized to ensure a balanced assessment.

### 2.4. Data Analysis

During the experiment, the x- and y-coordinates of the target marker and stylus tip were recorded at a sampling rate of 50 Hz and expressed in millimeters. Positional error was defined as the Euclidean distance between the target and stylus positions.

(1) $$Error[mm]=\sqrt{{\left(M_{x}-P_{x}\right)}^{2}+{\left(M_{y}-P_{y}\right)}^{2}}$$

In Equation (1), $M_{x}$ and $M_{y}$ are the x- and y-coordinates of the target marker, respectively, and $P_{x}$ and $P_{y}$ are the corresponding coordinates of the stylus tip. Because all coordinate values were expressed in millimeters, positional error was also expressed in millimeters.

For each participant, positional errors were averaged across the five trials separately for each speed and visibility condition. Thus, separate mean target-visible and target-invisible positional errors were obtained at the low, medium, and high speeds.

For each participant and each speed condition, the Tracer Error Ratio was calculated by dividing the mean target-invisible positional error by the corresponding mean target-visible positional error. Both mean errors were averaged across the five trials. Accordingly, three separate ratios, one for each speed condition, were obtained for each participant. A ratio of 1 indicates equal mean positional errors in the target-invisible and target-visible conditions. A ratio greater than 1 indicates a larger error in the target-invisible condition, whereas a ratio less than 1 indicates a smaller error. Thus, the ratio expresses the relative magnitude of positional error between the two visibility conditions as a single value. The ratios were calculated before group-level summary statistics were obtained and were analyzed separately from the positional-error data. The Tracer Error Ratio is dimensionless.

An a priori sample-size calculation was performed using G*Power software (version 3.1.9.7; Heinrich Heine University Düsseldorf, Düsseldorf, Germany) for a repeated-measures design with three groups, assuming a large effect size of f=0.40, an alpha level of 0.05, and a statistical power of 0.80. The estimated minimum total sample size was 36 participants, corresponding to at least 12 participants per group [26].

The positional-error data were analyzed using a 3 × 2 × 3 mixed repeated-measures analysis of variance, with Speed (low, medium, and high) and Visibility (target-visible and target-invisible) as within-subject factors and Age Group (younger children, older children, and adults) as the between-subject factor. To further examine the Visibility × Age Group interaction, separate 2 × 3 mixed repeated-measures analyses were conducted for each speed condition, with Visibility as the within-subject factor and Age Group as the between-subject factor.

The participant-level Tracer Error Ratios were analyzed separately using a 3 × 3 mixed repeated-measures analysis of variance, with Speed as the within-subject factor and Age Group as the between-subject factor. Three separate Tracer Error Ratios, corresponding to the low-, medium-, and high-speed conditions, were included for each participant.

Mauchly’s test was used to assess the sphericity assumption. When sphericity was violated, Greenhouse–Geisser-corrected degrees of freedom and *p*-values were reported. Box’s M test and Levene’s test were used to assess the homogeneity of covariance matrices and variances, respectively. Significant effects and interactions were followed by simple-effect or pairwise comparisons, as appropriate. Pairwise comparisons were adjusted using the Bonferroni method. Effect sizes were reported as partial eta squared ($\eta_{p}^{2}$), and statistical significance was set at p<0.05.

Only dominant-hand data were included in the statistical analyses.

## 3. Results

### 3.1. Overall Effects of Speed, Visibility, and Age Group on Positional Error

The positional-error data were analyzed using a 3 (Speed: low, medium, and high) × 2 (Visibility: target visible and target invisible) × 3 (Age Group: younger children, older children, and adults) mixed repeated-measures ANOVA.

Mauchly’s test indicated that the assumption of sphericity was violated for Speed (W=0.338, p<0.001) and Speed × Visibility (W=0.783, p=0.003). Therefore, Greenhouse–Geisser-corrected results were reported for these effects.

There were significant main effects of Speed, F(1.203,57.761)=429.679, p<0.001, $\eta_{p}^{2}=0.900$, and Visibility, F(1,48)=161.873, p<0.001, $\eta_{p}^{2}=0.771$. The main effect of Age Group was not significant, F(2,48)=1.970, p=0.151, $\eta_{p}^{2}=0.076$.

Significant interactions were observed for Speed × Visibility, F(1.644,78.907)=70.781, p<0.001, $\eta_{p}^{2}=0.596$; Speed × Age Group, F(2.407,57.761)=4.364, p=0.012, $\eta_{p}^{2}=0.154$; and Visibility × Age Group, F(2,48)=22.505, p<0.001, $\eta_{p}^{2}=0.484$. The Speed × Visibility × Age Group interaction was not significant, F(3.288,78.907)=0.509, p=0.694, $\eta_{p}^{2}=0.021$.

To further characterize the Visibility × Age Group interaction, separate analyses were conducted for each speed condition.

### 3.2. Speed-Specific Positional-Error Results

#### 3.2.1. Low-Speed Condition

**Figure 3:**
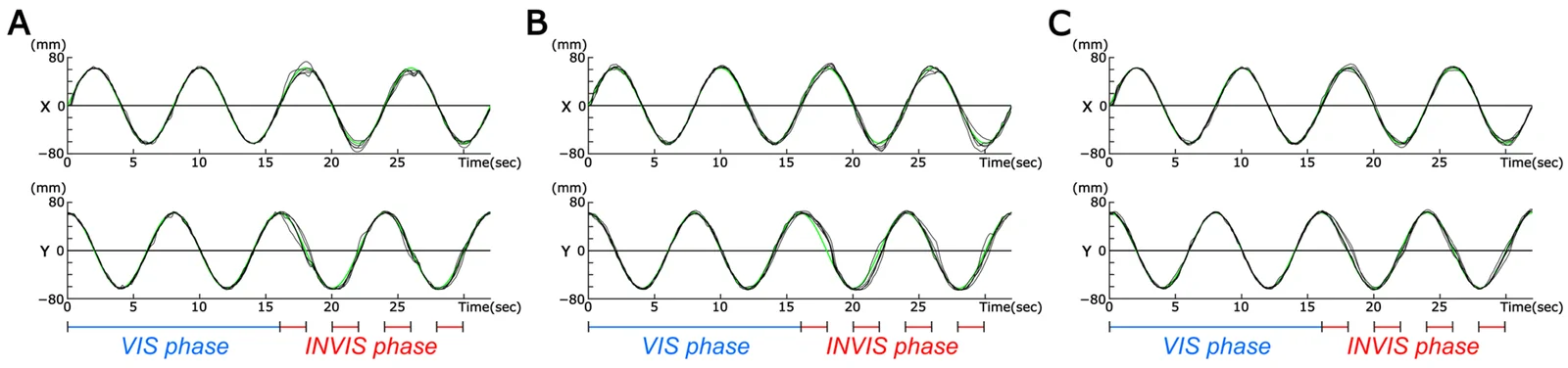
Representative target and stylus trajectories at low speed for (**A**) younger children, (**B**) older children, and (**C**) adults. The green line represents the target trajectory, and the black lines represent stylus trajectories from Trials 1–5. Blue and red horizontal bars indicate the target-visible (VIS) and target-invisible (INVIS) phases, respectively.

Representative target and stylus trajectories at low speed are shown in Figure 3. The mean target-visible positional errors were 3.640 mm in younger children, 3.417 mm in older children, and 3.618 mm in adults. The corresponding target-invisible positional errors were 10.044, 9.575, and 6.233 mm, respectively.

**Figure 4:**
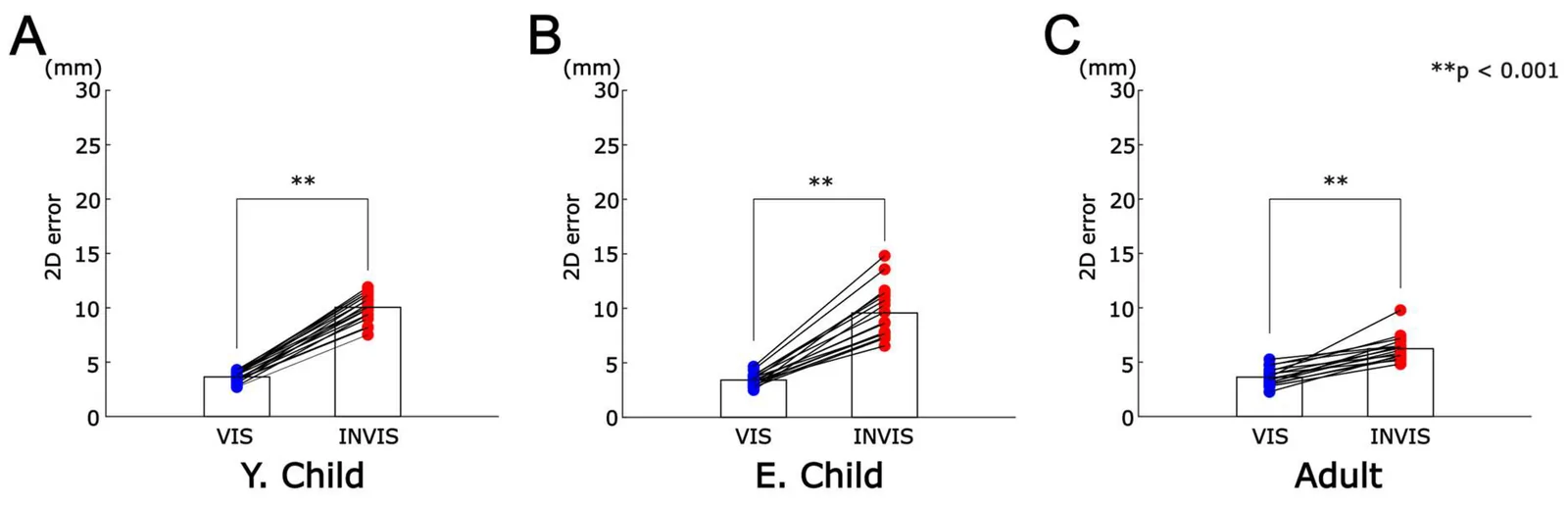
Positional errors under the target-visible and target-invisible conditions at low speed for (**A**) younger children, (**B**) older children, and (**C**) adults. Bars indicate group means; blue and red points indicate participant-level mean positional errors under the target-visible and target-invisible conditions, respectively. Lines connect paired values from the same participant. Values are reported in millimeters. Asterisks indicate statistically significant differences between the target-visible and target-invisible conditions, as shown in the figure (** *p* < 0.001).

At low speed, the Visibility × Age Group interaction was significant, F(2,48)=34.004, p<0.001, $\eta_{p}^{2}=0.586$. As shown in Figure 4, target-invisible positional error was significantly greater than target-visible positional error in younger children, older children, and adults (all p<0.001).

No significant age group differences were observed in the target-visible condition. In the target-invisible condition, adults had significantly lower positional errors than both younger children and older children (both p<0.001), whereas the two child groups did not differ significantly.

#### 3.2.2. Medium-Speed Condition

**Figure 5:**
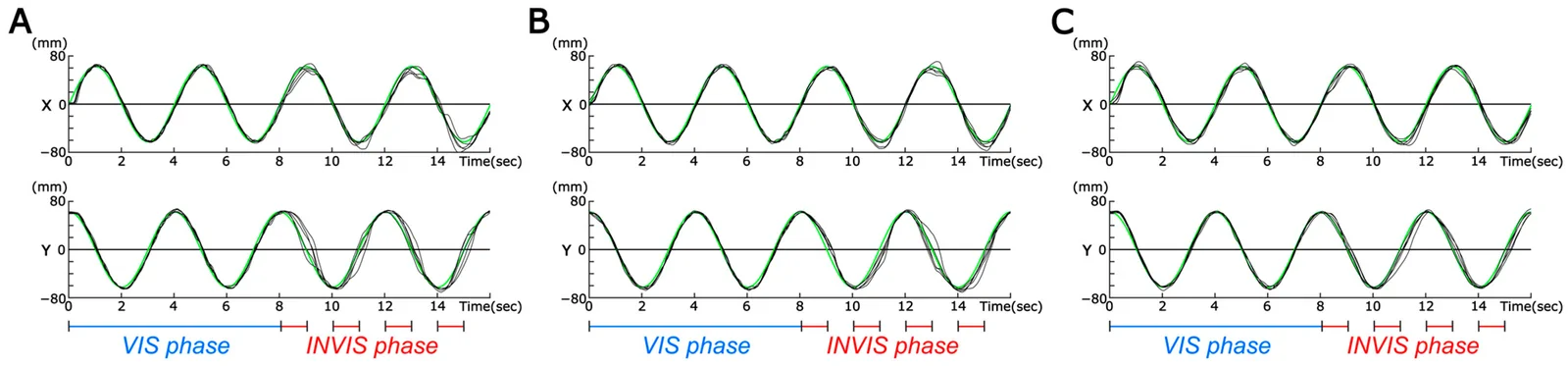
Representative target and stylus trajectories at medium speed for (**A**) younger children, (**B**) older children, and (**C**) adults. The green line represents the target trajectory, and the black lines represent stylus trajectories from Trials 1–5. Blue and red horizontal bars indicate the target-visible (VIS) and target-invisible (INVIS) phases, respectively.

Representative target and stylus trajectories at medium speed are shown in Figure 5. The mean target-visible positional errors were 6.767 mm in younger children, 6.305 mm in older children, and 7.322 mm in adults. The corresponding target-invisible positional errors were 11.716, 10.515, and 8.552 mm, respectively.

**Figure 6:**
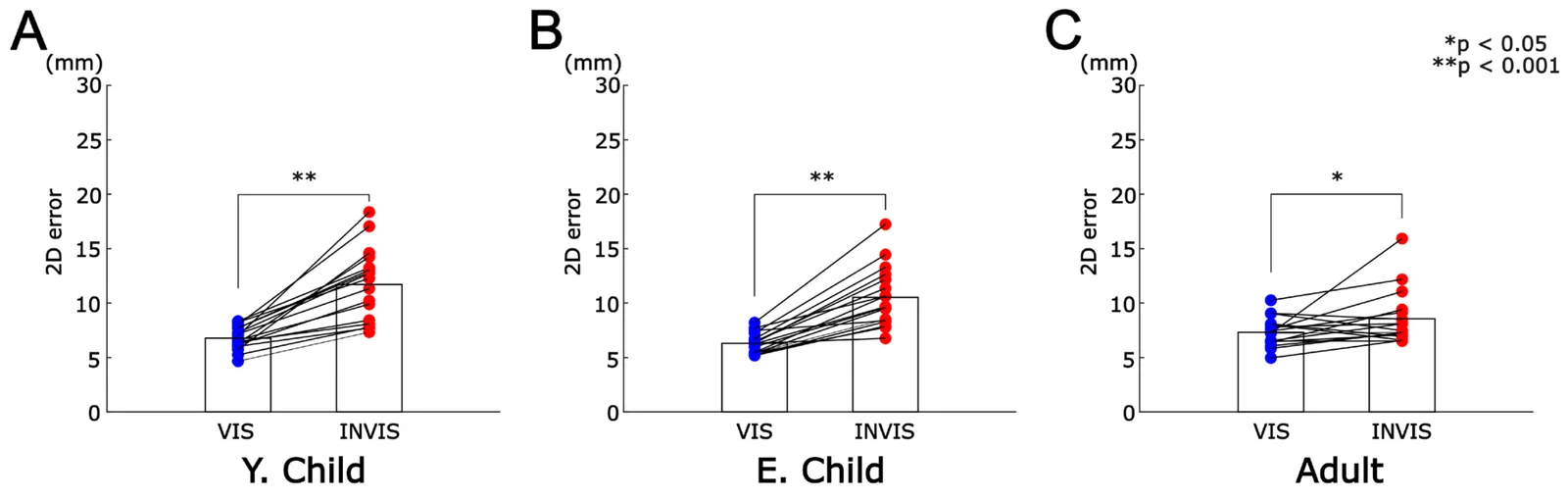
Positional errors under the target-visible and target-invisible conditions at medium speed for (**A**) younger children, (**B**) older children, and (**C**) adults. Bars indicate group means; blue and red points indicate participant-level mean positional errors under the target-visible and target-invisible conditions, respectively. Lines connect paired values from the same participant. Values are reported in millimeters. Asterisks indicate statistically significant differences between the target-visible and target-invisible conditions, as shown in the figure (* *p* < 0.05, ** *p* < 0.001).

At medium speed, the Visibility × Age Group interaction was significant, F(2,48)=10.426, p<0.001, $\eta_{p}^{2}=0.303$. As shown in Figure 6, target-invisible positional error was significantly greater than target-visible positional error in younger children and older children (both p<0.001). A smaller but statistically significant difference was also observed in adults (p=0.049).

In the target-visible condition, adults had significantly greater positional error than older children (p=0.032); the other age groups comparisons were not significant. In the target-invisible condition, adults had significantly lower positional error than younger children (p=0.007); the remaining comparisons were not significant.

#### 3.2.3. High-Speed Condition

**Figure 7:**
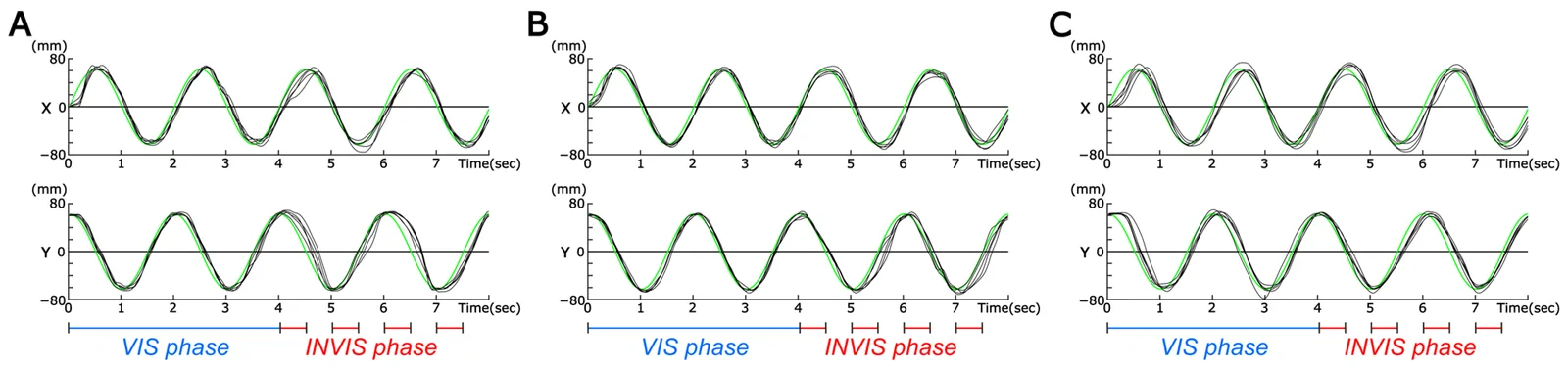
Representative target and stylus trajectories at high speed for (**A**) younger children, (**B**) older children, and (**C**) adults. The green line represents the target trajectory, and the black lines represent stylus trajectories from Trials 1–5. Blue and red horizontal bars indicate the target-visible (VIS) and target-invisible (INVIS) phases, respectively.

Representative target and stylus trajectories at high speed are shown in Figure 7. The mean target-visible positional errors were 15.191 mm in younger children, 13.951 mm in older children, and 17.108 mm in adults. The corresponding target-invisible positional errors were 17.783, 15.155, and 15.774 mm, respectively.

**Figure 8:**
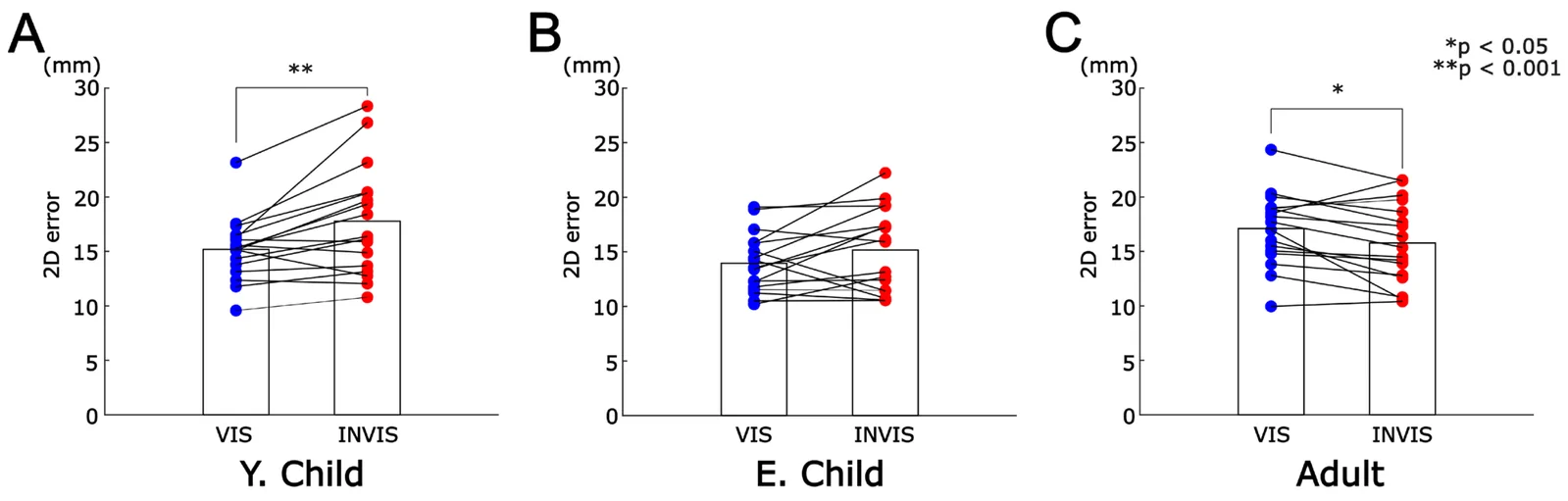
Positional errors under the target-visible and target-invisible conditions at high speed for (**A**) younger children, (**B**) older children, and (**C**) adults. Bars indicate group means; blue and red points indicate participant-level mean positional errors under the target-visible and target-invisible conditions, respectively. Lines connect paired values from the same participant. Values are reported in millimeters. Asterisks indicate statistically significant differences between the target-visible and target-invisible conditions, as shown in the figure (* *p* < 0.05, ** *p* < 0.001).

At high speed, the Visibility × Age Group interaction was significant, F(2,48)=9.351, p<0.001, $\eta_{p}^{2}=0.280$. As shown in Figure 8, target-invisible positional error was significantly greater than target-visible positional error in younger children (p<0.001). The difference was not significant in older children (p=0.071). In adults, target-invisible positional error was lower than target-visible positional error, and the within-group difference was statistically significant (p=0.046).

In the target-visible condition, adults had significantly greater positional error than older children (p=0.011); the other age group comparisons were not significant. No significant age group differences were observed in the target-invisible condition.

### 3.3. Tracer Error Ratio Analysis

The participant-level Tracer Error Ratios were analyzed separately using a 3 (Speed: low, medium, and high) × 3 (Age Group: younger children, older children, and adults) mixed repeated-measures ANOVA. Mauchly’s test did not indicate a significant violation of sphericity (W=0.881, p=0.051).

There were significant main effects of Speed, F(2,96)=302.388, p<0.001, $\eta_{p}^{2}=0.863$, and Age Group, F(2,48)=28.652, p<0.001, $\eta_{p}^{2}=0.544$. The Speed × Age Group interaction was also significant, F(4,96)=11.412, p<0.001, $\eta_{p}^{2}=0.322$.

The mean Tracer Error Ratio was progressively lower across the low-, medium-, and high-speed conditions in all three age groups. Younger children had mean ratios of 2.780, 1.729, and 1.161 at low, medium, and high speed, respectively. Older children had corresponding mean ratios of 2.818, 1.663, and 1.091, whereas adults had mean ratios of 1.779, 1.185, and 0.923.

Adults had significantly lower Tracer Error Ratios than both younger children and older children at low speed (both p<0.001), medium speed (p<0.001 and p=0.001, respectively), and high speed (p=0.001 and p=0.019, respectively). No significant differences were observed between the two child groups at low or medium speed (both p=1.000) or at high speed (p=0.719).

The estimated means and 95% confidence intervals were 2.780 (2.553–3.007), 1.729 (1.556–1.901), and 1.161 (1.077–1.245) for younger children; 2.818 (2.592–3.045), 1.663 (1.491–1.835), and 1.091 (1.007–1.175) for older children; and 1.779 (1.553–2.006), 1.185 (1.013–1.357), and 0.923 (0.839–1.007) for adults at low, medium, and high speed, respectively.

At high speed, the mean Tracer Error Ratio in adults was below 1, although its 95% confidence interval included 1.

Table 2 summarizes the mean target-visible and target-invisible positional errors and the mean participant-level Tracer Error Ratios for each age group and speed condition.

## 4. Discussion

The present study examined two-dimensional circular target tracking performance across three age groups, three target speeds, and two target-visibility conditions using a portable tablet–stylus system. Adults exhibited lower ratios than both child groups at all three speeds, whereas no significant differences were observed between the younger- and older-child groups.

These findings indicate an adult–child difference in the relative behavioral effect of target disappearance. However, they do not demonstrate a continuous developmental progression from younger children to older children and then to adults. Moreover, because the present study measured behavioral tracking errors rather than neural activity, the results do not directly identify a specific cerebellar mechanism.

### 4.1. Positional-Error Differences Across Speed and Visibility Conditions

Positional error increased substantially with increasing target speed under both target-visible and target-invisible conditions. The significant Speed × Visibility interaction further indicated that the difference between the two visibility conditions varied across the speed conditions.

At low speed, target-invisible positional error was significantly greater than target-visible positional error in all three age groups. No age group differences were observed under the target-visible condition, whereas adults showed lower target-invisible errors than both child groups. Thus, the principal adult–child difference at low speed emerged when current target-related visual information was unavailable.

At medium speed, target-invisible positional error remained greater than target-visible positional error in all three groups, although the difference was smaller in adults. Adults showed greater target-visible error than older children but lower target-invisible error than younger children. The other age group comparisons were not significant. Therefore, the results do not indicate a uniform adult advantage across all medium-speed comparisons; rather, the age group differences depended on both the visibility condition and the specific groups compared.

At high speed, target-invisible error remained significantly greater than target-visible error in younger children, whereas the difference was not significant in older children. In adults, target-invisible error was slightly but significantly lower than target-visible error. Adults showed greater target-visible error than older children, while no age group differences were found under the target-invisible condition. The absence of target-invisible age group differences at high speed indicates that the adult–child differences observed at lower speeds were no longer evident under the highest-speed condition.

The target-visible and target-invisible conditions primarily differed in the availability of current target-related visual information. When the target was visible, participants could continuously compare the target and stylus positions. During target disappearance, current information about the target position and target–stylus discrepancy was unavailable; however, previously observed target motion, learned spatiotemporal regularities of the repeated circular trajectory, and sensory information related to the participant’s own movement could remain available. Accordingly, target disappearance increased the relative demand on predictive tracking but did not isolate a purely feedforward process.

### 4.2. Interpretation of the Tracer Error Ratio

The Tracer Error Ratio was calculated separately for each participant and speed condition by dividing the mean target-invisible positional error by the corresponding mean target-visible positional error. It therefore represents a derived behavioral comparison between the two target-visibility conditions. It should not be interpreted as a direct physiological measure of feedback–feedforward weighting, cerebellar function, or internal model efficiency.

The mean Tracer Error Ratio was progressively lower across the low-, medium-, and high-speed conditions in all three age groups. This pattern partly reflects the relatively greater increase in target-visible positional error as speed increased. However, because the speeds were always administered in a fixed low-to-medium-to-high sequence, the observed differences cannot be attributed exclusively to target speed. Practice, adaptation, or fatigue associated with the fixed sequence may also have contributed.

Adults exhibited lower ratios than both child groups at every speed, whereas the younger- and older-child groups did not differ significantly. These findings indicate that the relative behavioral effect of removing continuous target-related visual information differed between adults and children, but they do not support a stepwise developmental progression between the two child groups. At high speed, the adult mean ratio was below 1, although its 95% confidence interval included 1. This group estimate should therefore not be interpreted as definitive evidence that target disappearance generally improved tracking performance in adults.

Previous studies indicate that feedback responses and feedforward commands can interact and may share learned representations of limb dynamics [6,27,28]. Internal model theories also propose that forward models predict the consequences of motor commands and may supplement sensory feedback when that information is delayed or incomplete [10,11,13,15]. Cerebellar processing has additionally been proposed to contribute to prediction of future motor states [29]. These theories provide a conceptual framework for discussing predictive motor control, but they do not validate the Tracer Error Ratio or establish that the present age group differences resulted from the maturity of a specific cerebellar internal model.

### 4.3. Relation to Previous Tracking Studies

The present study combines elements from several related lines of tracking research. Temporarily invisible-target paradigms have been used to investigate predictive representations of target motion during ocular tracking [9]. Developmental changes in anticipatory control have also been demonstrated in a dynamic manual tracking task performed with an electronic pen [8]. The present study differs from both approaches by comparing tablet–stylus positional errors across target-visible and target-invisible conditions, three target speeds, and three age groups. Neither previous study directly validated the Tracer Error Ratio used in the present analysis.

Previous work has also examined error correction and proactive responses during mutual or externally guided hand tracking [16,17], and circular wrist-tracking studies have characterized position-, direction-, and speed-related tracking errors [18]. These studies support the broader use of tracking tasks to examine sensorimotor control, but their task structures and outcome measures differ from those used here.

Age-related differences in endpoint kinematics, visuomotor coordination, and upper limb movement have been reported in school-aged children and adults [23,24,30]. Tracing-task research has likewise demonstrated effects of age on visuomotor performance [31]. The adult–child differences observed in the present study are broadly consistent with this literature. Nevertheless, the absence of significant TER differences between the two child groups indicates that the current results should not be described as evidence of a continuous age-related trajectory across all three groups.

Developmental studies have also shown that children and adults may differ in their use of anticipatory and corrective motor control processes [7,8]. However, the postural-disturbance paradigm used by Hay and Redon [7] and the accelerating target task used by van Roon et al. [8] differ substantially from the present circular tracking paradigm. These references therefore provide indirect developmental context rather than direct validation of the present visibility manipulation or TER.

### 4.4. Relevance to Sensor-Based Human Motion Analysis

The tablet–stylus system provided a relatively simple method for acquiring two-dimensional target and stylus coordinates. The task also allowed participant-level positional errors and paired target-visible–target-invisible changes to be visualized.

From a sensor-based human motion-analysis perspective, the present task may provide a practical research platform for investigating upper limb tracking behavior. Its simple setup may facilitate repeated measurements in research environments where conventional motion-capture systems are impractical. However, the study included only healthy school-aged children and young adults and did not establish clinical validity, diagnostic accuracy, or sensitivity to rehabilitation-related change.

Previous research has reported visuomotor performance differences between typically developing children and children with motor coordination difficulties [32]. Such findings provide a rationale for evaluating sensor-based tracking tasks in broader populations, but they do not establish that the present task or TER is clinically useful. Studies involving patient groups and comparisons with established motor assessment instruments are required before clinical or rehabilitation applications can be claimed.

The platform may also have future relevance to human–computer interaction and motor-training research, although such applications require further validation of reliability, learning effects, usability, and responsiveness.

### 4.5. Limitations and Future Directions

Several limitations should be considered. First, the target-visible and target-invisible conditions did not isolate pure feedback and feedforward control. During target disappearance, participants could continue to use previously observed target motion, the repeated circular trajectory, learned temporal regularities, and sensory information related to their own movement. The target-invisible condition should therefore be interpreted as imposing a greater relative predictive-tracking demand rather than as a purely feedforward condition.

Second, target speeds were presented in a fixed low-to-medium-to-high order. Speed was consequently confounded with measurement order, and the observed differences cannot be fully separated from potential practice, familiarization, adaptation, or fatigue effects. Future studies should incorporate standardized familiarization and, where feasible, counterbalanced measurement orders while ensuring that participants receive sufficient prior exposure to the target trajectory.

Third, the first two cycles of each trial were analyzed as the target-visible condition and preceded the cycles containing target-invisible segments. These cycles were not warm-up or practice trials; they constituted the continuously visible data condition. Nevertheless, their fixed position before the target-invisible cycles may have introduced carryover from the visible to the invisible portions. Future protocols should examine methods of providing equivalent prior trajectory exposure while reducing systematic order effects.

Fourth, the Tracer Error Ratio is a derived relative-error index whose measurement properties have not been established. The present study did not evaluate convergent validity against established motor development measures, test–retest reliability, measurement error, minimum detectable change, responsiveness, or validity in patient populations. These properties require evaluation before the ratio can be used as a clinical or longitudinal outcome.

Fifth, the sex distribution was unequal across the age groups. The study was designed primarily to examine age group differences rather than sex-related effects, and the small and uneven age-by-sex cells did not support a reliable sex-stratified analysis. The possible contribution of sex to the observed group differences therefore cannot be excluded.

Sixth, although both hands were tested, only dominant-hand data were included in the final analysis. Because hand side and movement direction were coupled in the analyzed dataset, their separate effects could not be determined. Future studies should examine hand and movement direction effects independently.

Finally, positional error was defined as the two-dimensional Euclidean distance between the target and stylus positions. This endpoint measure does not fully characterize the temporal and kinematic features of tracking performance. Future work could combine positional error with jerk, joint kinematics, or other sensor-derived measures to provide a more comprehensive characterization of motor performance.

## Figures and Tables

**Table 1:** Demographic Information of Participants.

No	Lower-Age Elementary Child			Higher-Age Elementary Child			Adult		
	Gender	Age	Dominant Hand	Gender	Age	Dominant Hand	Gender	Age	Dominant Hand
1	M	10	R	F	11	R	F	21	R
2	F	8	R	F	11	R	F	21	R
3	M	8	R	F	11	L	F	21	R
4	F	9	L	F	12	R	M	21	R
5	M	10	R	F	11	R	M	21	R
6	F	8	L	M	11	R	F	22	R
7	M	10	L	M	11	R	F	21	R
8	F	8	R	F	12	R	F	21	R
9	F	8	R	F	13	R	M	21	R
10	M	10	R	M	12	R	F	22	R
11	F	9	R	F	11	R	M	30	R
12	F	10	R	F	12	R	M	20	R
13	F	10	L	M	12	R	M	20	R
14	F	9	R	M	12	R	M	26	L
15	F	10	R	F	13	R	M	30	R
16	F	10	R	F	11	R	M	23	R
17	F	8	R	F	11	R	M	24	R

**Table 2:** Positional errors and Tracer Error Ratios by age group and speed condition.

Age Group	Speed	Target-Visible Positional Error [mm]	Target-Invisible Positional Error [mm]	Tracer Error Ratio (INVIS/VIS)
Younger children	Low	3.640	10.044	2.780
Younger children	Medium	6.767	11.716	1.729
Younger children	High	15.191	17.783	1.161
Older children	Low	3.417	9.575	2.818
Older children	Medium	6.305	10.515	1.663
Older children	High	13.951	15.155	1.091
Adults	Low	3.618	6.233	1.779
Adults	Medium	7.322	8.552	1.185
Adults	High	17.108	15.774	0.923

Note: Positional errors are group means and are reported in millimeters. Tracer Error Ratios were calculated separately for each participant at each speed as target-invisible/target-visible positional error and then averaged within each age group.

## Data Availability

The datasets generated during and/or analyzed during the current study are available from the corresponding author on reasonable request.

## Supplementary Materials

The following supporting information can be downloaded at: https://www.mdpi.com/article/10.3390/s26175344/s1, Figure S1: Schematic representation of the feedback and feedforward motor-control framework used to interpret target-visible and target-invisible tracking conditions.
